# Supplementary figures and images for: Impact of the ABxSG Mobile Application on Antibiotic Prescribing: An Interrupted Time Series Study
Source: Antibiotics (Basel). 2025 Sep 16;14(9):933. doi: 10.3390/antibiotics14090933 (PMC12466656; doi:10.3390/antibiotics14090933)

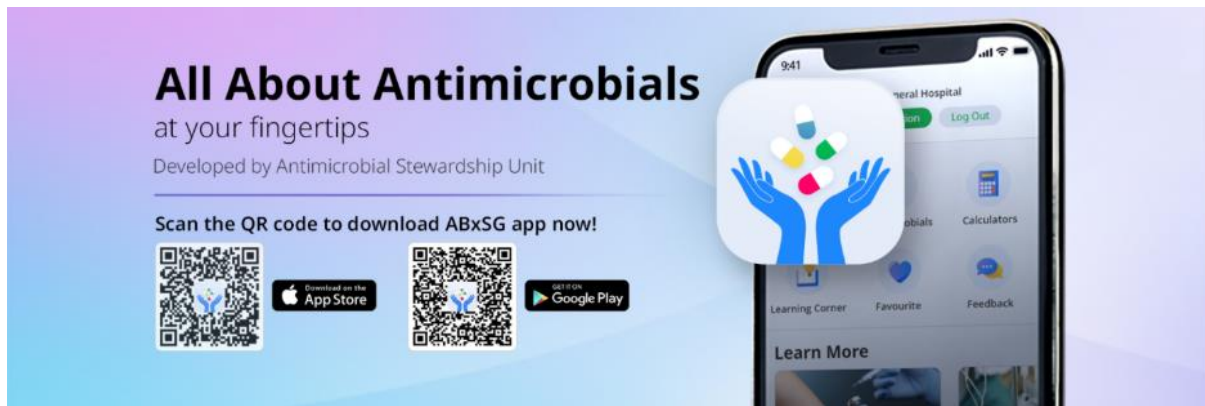

**Figure S1.** Intranet banner

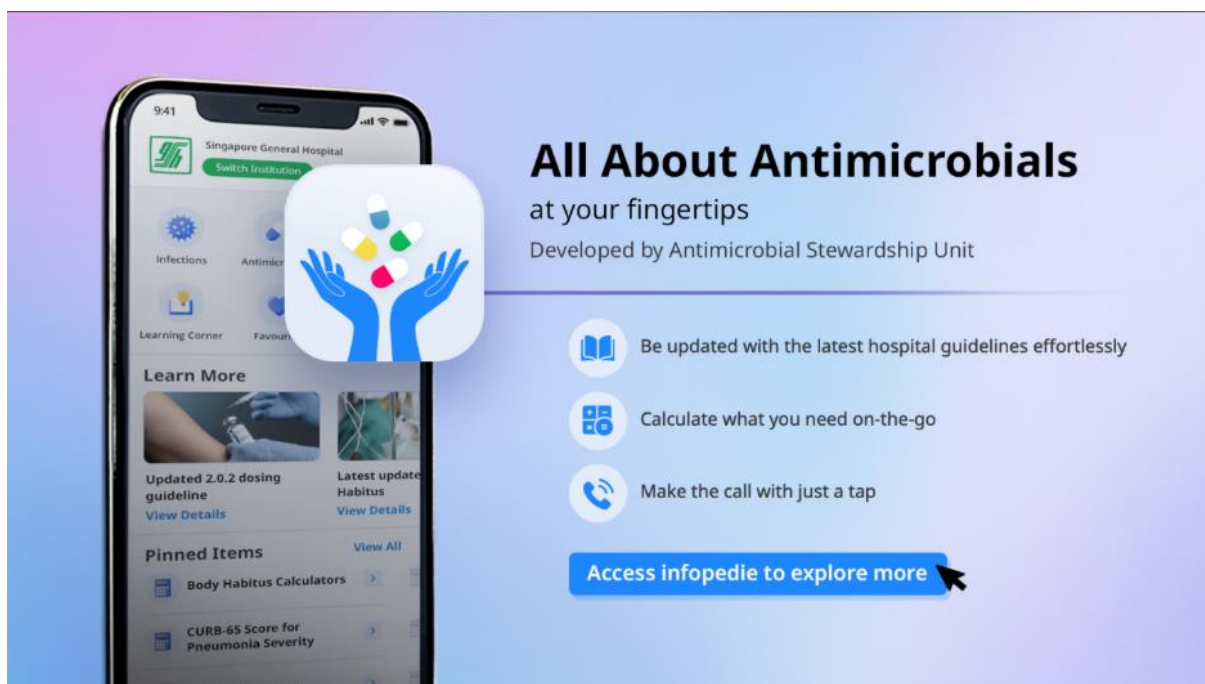

**Figure S2.** Screensaver

Supplement: Supplementary file 1 [file antibiotics-14-00933-s001.zip › antibiotics-3836875-supplementary.pdf]
